# Lignocellulose, dietary fibre, inulin and their potential application in food

**DOI:** 10.1016/j.heliyon.2022.e10459

**Published:** 2022-08-29

**Authors:** Oyekemi Olabisi Popoola-Akinola, Temiloluwa Joy Raji, Babatunde Olawoye

**Affiliations:** Department of Food Science and Technology, First Technical University, P.M.B. 5015 Ibadan, Oyo State, Nigeria

**Keywords:** Lignocellulose, Indigestible polysaccharide, Inulin, Functional ingredients

## Abstract

In recent years, due to food insecurity, lignocellulose, dietary fibre as well as inulin have received wider attention owing to their abundance and being relatively low-cost indigestible polysaccharides. Since the recognition, acceptance of the consumption and utilization of these polysaccharides, as well as their attraction in science and industry has grown tremendously. There have been further researches carried out to ascertain the fact that people who consume or utilize these polysaccharides have low exposure to some fatal life-threatening illnesses. Rich sources of indigestible polysaccharides such as vegetables, cereals, fruits and nuts are beneficial to good health as consuming them reduce the occurrence of degenerating diseases such as colon cancer, heart disease, diabetes, etc. Despite these increasing facts depicting their advantages in the state of human health, their intake and utilization still fall below the acceptable limit and the knowledge of how they work in the human body are minimal with their explicit actions not easily shown. Hence, this review gives a better understanding of the significance of lignocellulose, dietary fibre and inulin, their functions, classifications, types and applications in the food industry, thereby exposing their various uses as these polycarbohydrates were considered a waste before now.

## 1 Introduction

Polysaccharides are biomolecules consisting of chains of monosaccharides such as but not limited to glucose, galactose, fructose, xylose, mannose etc., and are joined together by glycosidic bonds. The classes of polysaccharides include cellulose, which gives cellular support to plant, starch and glycogen which form the storage polysaccharides for storing energy while glycoconjugates which are formed by the covalent bonding of polysaccharides to lipids (glycolipids) or proteins (glycoproteins) are responsible for cellular communication of polysaccharides sending signals within and between cell (Gopinath et al., 2018). Nutritionally, starch, cellulose as well as glycogen are the principal polysaccharides. Starch acts as an energy source derived from plants while glycogen forms the main carbohydrate storage in the liver and muscles of humans and is converted to glucose as needed. Starch is employed in many industrial applications such as in the food industry, for thickening, emulsifying, gelling, clouding and binding. Cellulose on the other end is a composite carbohydrate which forms the components of the cell wall in majority of plants (Lee and Won, 2000) and it has found application in vast products such as textiles, paper, pharmaceuticals in addition to explosives (Gupta et al., 2019). According to researches, polysaccharides differ when undergoing digestion in the human gastrointestinal tract; some digest while others do not. Owing to this, polysaccharides are classified based on their digestible status in humans' gastrointestinal tracts into two major classes; digestible and indigestible polysaccharides. In human diets, a part of plant material that resists digestion is usually classified as dietary fibre (Dhingra et al., 2012). In recent times, novel products, processes and applications aimed at promoting the utilization of these indigestible polysaccharides, as food and functional food ingredient are being promoted by industries to enable consumers appropriately increase their consumption of dietary fibre. Salovaara et al. (2007) reported that there has been the realization of the multifunctionality of bioactive carbohydrates, which resulted in a wide development of potential consumer market for food products and ingredients rich in fibre. One major source of fibre in the food industry is through crops and food waste, however, in recent years, there has been a trend to source fibre from feed and non-food products. In this present study, various types and source of indigestible polysaccharides and their application in the food industry have been reviewed.

## 2 Classification of polysaccharides

### 2.1 Digestible polysaccharides

The term ‘digestible’ refers to those polysaccharides that can be digested by humans in the sense that enzymes in the human stomach can break them down. These digestible classes include starch, glycogen, etc. They are usually made as foods for humans and they consist of hundreds of glucose units linked together by acetal linkages. The human body is modified in alpha form, making it easier for the body to break them down and the breaking down of these polysaccharides is because the human body can digest glucose in its simplest form and polysaccharides are made of hundreds of glucose linked together by bonds.

### 2.2 Classification of digestible polysaccharides

#### 2.2.1 Starch

Starch, a polymeric macromolecule made up of repeating units of glucose is the most regularly occurring digestible polysaccharide. Starch exists in two d-glucose polymers which are amylose and amylopectin, with both being polymers of d-glucose. Amylose polymer of d-glucose is made up of a linear unbranched chain with the residues of glucose attached exclusively through α 1–4 glycosidic bond. Amylopectin constitutes a polymer chain that is branched with the branching occurring through α 1–6 glycosidic bond. Of the total content of starch, a percentage of 15–20% and 80–85% is constituted by amylose and amylopectin respectively. During the process of digestion, starch is broken down in the gastrointestinal tract into a unit of glucose, when this glucose enters the blood stream, it resulted in hyperglycemia (a result arises from high sugar content in the blood stream). Starch is mainly found in starchy vegetables such as sweet potato, potatoes, corn, grains, legumes winter squash and other types of vegetables.

#### 2.2.2 Glycogen

In animal tissues, glycogen forms the main form of carbohydrate that is stored and is primarily located in the skeletal muscles and liver. Just like amylopectin, it consists of a polyglucose molecule that is highly branched, a reason for its comparison with starch. The residues of glucose within glycogen are useful as an easily available source of glucose. The degree of branching which is high in amylopectin and glycogen gives an essential metabolic benefit because it offers a great number of non-reducing ends where residues of glucose can be connected. The demands of energy by the body enzymatically removes glucose residues from non-reducing ends of the chains of glycogen and then enters the metabolic pathway which releases energy in a process known as glycogenolysis.

### 2.3 Indigestible polysaccharides

Indigestible polysaccharides are complex parts of polysaccharides which comprises glucose, linked together by a β-1,4-glycosidic bond that cannot be digested by humans. This means that the enzymes responsible for digestion cannot act or utilize them. They form the structural and protective parts of plants. Some animals can digest them but not directly, as these animals possess symbiotic bacteria in their small intestines, which utilizes these foods, breaking them down for further digestion processes. Indigestible polysaccharides contain all the long-chain carbohydrates that cannot be digested, whether part of plants or by-products of plants. Indigestible polysaccharides include lignocellulose (cellulose, hemicellulose, lignin and pectin), dietary fibre (gum, resistant starch, agar) and inulin. For example, resistant starch is an indigestible polysaccharide, though not a dietary fibre, but it functions like one (Kris, 2018). As generally known, the most common polysaccharide is starch and the carbohydrate in our food is present in the form of starch, but it has been realized that not all these starches get broken down in the digestive tract. Some go through the digestion process unchanged, meaning they are not broken down into sub-components for utilization. These types of starches are referred to as resistant starch, as they are immune to digestion processes (Ferguson et al., 2001). These resistant starches are important in our diet because they reduce the blood sugar level. After all, when starch is broken down, it is reduced to glucose, which is in the form of sugar, supplying sugar to the blood. Therefore, eating a high quantity of starch results in a high level of glucose in the human blood, however, in the case of consuming resistant starch which is immune to digestion, there is a reduction in the glucose in the blood because the starch is not broken dow thereby reducing the risks of some blood diseases that might arise due to excess glucose in the blood (Olawoye et al., 2020b).

## 3 Lignocellulose

Lignocellulose refers to a class of indigestible polysaccharides that constitute majorly the cell wall of plant materials such as wheat straw, dead branches, corn stover, fallen grasses, leaves and wood chips. In plants, the total amount of lignocellulose accounts for 30–50% in respect to the entire dry weights. They are renewable, widely available and relatively low-cost polysaccharides, which include a complex mixture of carbohydrate polymers, cellulose and hemicellulose covalently bound to lignin (an aromatic abundant polymer) as well as pectic substance. Lignocellulose resists biodegradation and gives stability as well as structural vigour to the plant cell wall. This structural vigour is attributed to the cellulose and hemicellulose cross-linking to lignin (an aromatic polysaccharide) via ether and ester linkages resulting in a highly complex structure. Like other polysaccharides, monomers of lignocellulose are linked together by glycosidic bonds and are found abundantly in agricultural and food wastes as shown in Table 1. Due to the inherent characteristics of lignocellulose such as a high level of cellulose crystallinity and strong layers of lignin, which limits its use in food, paper, energy and plastic industry, there is a need for pre-treatment for extractability of its component (cellulose, hemicellulose and lignin).

**Table 1:** Lignocellulose.

Nutrient	Monomers	Linkage	Source
Cellulose	Glucose	β-(1–4)	Fruits, vegetables (generally in plants), cereals
Hemicellulose	arabinose, mannose, galactose, glucose, xylose and uronic acid	β-(1–4) glycan	Cereals, bran, timber, legumes, rice husk, wheat straw
Lignin	p-coumaryl alcohol, coniferyl alcohol, and sinapyl alcohol	β-O-4 aryl ether bond	Fruit stones, vegetables (filaments of the garden bean), cereals, soft and hardwood
Pectin	Galacturonic	α-(1→4)-glycosidic	In the skin of fruits (majorly apples, quinces), vegetables

Each of the components of hemicellulose shows distinct chemical behaviour and due to its composite nature, the processing of lignocellulose could be challenging. Recalcitrance is a term used for the resistance of hemicellulose to degradation or separation. A combination of chemicals, heat, microorganisms and enzymes are needed to surmount this recalcitrance behaviour (1,2,3,4). The biomass of lignocellulose can broadly be classified into virgin biomass, waste biomass as well as energy crops. The manufacture of waste biomass is usually as a low-quality end product of several industrial systems such as forestry (paper mill and sawmill discards) and agriculture (straw, sugar cane bagasse, straw, etc.). Virgin biomass encompasses all terrestrial plants that are naturally occurring such as grass, bushes and trees. Energy crops such as Elephant grass and switchgrass have a lignocellulose biomass high yield for the production of biofuel (second generation).

### 3.1 Structure of lignocellulose

#### 3.1.1 Cellulose

Cellulose is a straight-chain polysaccharide that is made up of a linear glucan chain connected together by β-1,4-glycosidic bonds with cellobiose residues being the repeating unit at different degrees of polymerization (DP) and packed into microfibrils which are held together by intramolecular hydrogen bonds as well as intermolecular van der Waals forces (Bai et al., 2019). It is responsible for the strong outer covering of plants. It is a major covering protecting the inner tissues and cells of plants which helps plants to remain strong while growing and also throughout their span of life (Gupta et al., 2019). It may also be referred to as the backbone of plants.

It is indigestible to humans making it extremely important in the human diet. Even though humans cannot digest cellulose, some animals e.g., cows, sheep, etc. can digest cellulose due to the presence of bacteria in their stomachs. Also, these categories of animals can regurgitate, making cellulose digestible due to the long storage in their stomach. The beta acetal linkage is one major difference distinguishing cellulose from starch and this difference is responsible for its indigestibility in humans. Humans cannot digest cellulose because when humans eat, their body is made up of different enzymes, which act on the food to break them down for effective utilization. Therefore, the appropriate enzyme to break down the beta bond linking the glucose chain together in cellulose is lacking in humans, making it difficult for digestion (Charles, 2003). Despite the disadvantage of not being digested, cellulose acts as a mechanism that eases the transportation of food in the digestive tract. Other types of animals possessing this enzyme to digest glucose are termites. Also, cellulose possesses hydrogen bonding within the microfibrils and has poor solubility in most solvents. A very common industrial utilization of cellulose is in the production of paper and cotton. Since it is a friendly and not a poisonous polymer, it uses spread across different fields e.g., textile, food industry, cosmetics, nanotechnology, pharmaceutical industry etc. It is mostly used due to its adhesive properties as a binder in most products. Also, due to its fermentation properties, it is used in the production of ethers, ethanol and acetic acid, etc (Gupta et al., 2019). Some derivatives of cellulose include; celluloid, cellophane, rayon, cellulose acetate, nitrocellulose, methylcellulose, microcrystalline cellulose and ethulose. Cellulose strands are usually incorporated into but not attached covalently to the component of lignin-hemicellulose.

#### 3.1.2 Hemicellulose

Hemicellulose consists of branched polysaccharides (Ferguson et al., 2001) and can be likely said to be a support to cellulose. It has a short chain linked together when compared to cellulose. The major and distinct difference between cellulose and hemicellulose is that while cellulose is entirely made up of glucose, hemicellulose consists of different sugars coming together to form a unique compound. For instance, it can be made up of pentose; five (5) carbon atoms sugars e.g., xylose, arabinose and hexose sugars e.g., mannose, galactose etc. Xylose is always in higher proportion in the building up of hemicellulose compared to the composition of other sugars. Also, sugar alcohols can be found in the make-up of hemicellulose e.g., glucuronic acid. They are present in the soluble and insoluble forms of plant food materials (Hu et al., 2009). They increase stool hydration resulting from a proper movement of bowels and also prevent cholesterol absorption in the gut (Mudgil et al., 2012). Nuts, fruits, vegetables and legumes contain hemicellulose making up one-third of dietary fibre. Arabinoxylans, mannans and arabinogalactans are examples of hemicellulose.

##### 3.1.2.1 Arabinoxylan

Cereals like sorghum, barley, rye, oat, wheat and rice are composed of arabinoxylans which constitute a vital non-starch component of the cell wall of plants (Fincher and Stone, 1986). Arabinoxylans are composed of the thin walls of cells surrounding the aleurone layer and starchy endosperm of cereals. Cereal structures of arabinoxylans are made up of arabinose in addition to xylose having their molecular structure consisting of linear β-(1→4)-linked units of xylose. In the production of products such as grain spirits from wheat, arabinoxylans may pose problems as it forms aqueous solutions which are viscous (Sinha et al., 2011). The degree to which arabinose is substituted will be an influence on the adopted conformation and hence the viscosity of the solution. Arabinoxylans that are present in cereal grains are water-insoluble.

##### 3.1.2.2 Mannans

Mannans are polysaccharides, which are highly dense, water-insoluble and constitute the basis of plant hardiness. They are present in red and green algae displaying microfibrillar morphology and polymer structure in them (Ebringerová, 2005). Galactomannans and glucomannans are two divisions of mannan. Galactomannans are present as reversed polysaccharides in the endosperm of legumes and are soluble in water as well as take in water from the seeds of the endosperms. The ratio of mannose to galactose influences the viscosity and solubility of galactomannans. In addition, there can be considerable considerations in substituents distribution affecting the physical characteristics of galactomannans (Daas et al., 2000). Guar and locust beans, which are isolated from *Cyanosis tetragonolous* and *Ceratonia siliqua* respectively contain galactomannans. Cereal grains contain a small number of glucomannans which plays the role of storage in specific annual plant seeds such as irises and a certain number of lilies (Meier and Reid, 1982). They are also present in roots, tubers and bulbs of different plant types. Glucomannans, a plant-derived polysaccharide which belongs to the mannan family is the main component of *Amorphophallus konjac* (elephant yam or konjac plant). Glucomannan is a water-soluble heteropolysaccharide consisting β-(1–4)-linked d-glucose and d-mannose monomers in the ratio which differs according to source. However, glucomannan from konjac has a glucose to mannose molar ratio of 1:1.6. Aside their difference in molar ratio, diverse glucomannan also differs in the degree of acetylation. Konjac glucomannan can interact with many other polysaccharides, such as xanthan, carrageenan and agar, and forms thermally reversible gels which in turns made them useful in dairy and meat products as fat replacers.

##### 3.1.2.3 Arabinogalactans

In the walls of plant cells, arabinogalactans occur as Type I and Type II. Type 1 contains β-(1→4)- backbone of galactose, which is substituted with arabinose (Cheetham et al., 1993) while Type II contains β-(3,6)-polymer of galactose connected with arabinose. Type I and Type II arabinogalactans occur in legumes and rapeseed respectively. Compared to Type I which is a cell wall structural component, Type II is said to be connected with plasmalemma and extracellular space. There has been the isolation of Type II arabinogalactans from wheat flour (Fincher and Stone, 1974).

#### 3.1.3 Lignin

It is a complex non-carbohydrate aromatic and most abundant polymer after cellulose which is present in all wood. Commonly referred to as wood fuel, it occurs in plant cell walls and utilized in paper and paper by-products as well as lignocellulosic industries. It is a polyphenolic biomolecule whose primary monomer unit is made up of coniferyl alcohol, cinnamyl alcohol para-coumaryl alcohol and sinapyl alcohol. These monomers are linked together by dehydrogenative polymerization resulting in a 3-dimensional macromolecule with a multitude of C–C and ether-linked compounds. It binds to hemicellulose covalently forming around the polysaccharide a protective barrier against microbial invasion and oxidative stress. Lignin content differs according to plants and hence, essential parameters affect the polysaccharide decomposition efficiency as well as the glass transition temperature. It is majorly used as a natural resource in the production and extraction of fuel. In the petroleum industry, all resources that yield oil is used and lignin from plants is one of them. Researches have made it known that it is currently almost exclusively used for generating energy (Solihat et al., 2021). It is also one of the structural components of the plant responsible for its rigid shape and the part of the plant that joins the cellulose and hemicellulose which strengthens the cell wall. It serves as a non-susceptible part preventing the invasion of the plant body from pests and diseases. The introduction of lignin into the petroleum industry is because it is a part of the plant that is eco-friendly and it emits carbon dioxide which helps the growth of other plants. Before the discovery of lignin, products of petroleum derivatives depend on the organic breakdown of crude oil, but carbon in lignin (due to its aromatic compounds) is currently substituted and used to produce derivatives of petroleum and its by-products. Another potential use of lignin in the agro-industry is in the production of bioactive and therapeutic compounds which can be used in the treatment of many degenerative diseases. Chemically, lignin is composed of heterogenous polyphenolic and biopolymeric compounds and the degradation of lignin by white-rot fungi (basidiomycetes) yielded polyphenols and other compounds with therapeutic values such as antioxidant, anticancer/tumour, antimicrobial, as well as anti-diabetic properties.

#### 3.1.4 Pectin

Pectin is a structural anionic heteropolysaccharide and it is found abundantly in cells and tissues of plants. It is relatively cheap and non-toxic, making it a potential additive in food processing industries. It is mainly occurs in the middle lamella of plant cell walls as magnesium and calcium salt. The exact structure of pectin is yet unknown due to its complex structure which varies according to plants, maturity as well as storage. However, pectin generally has a homogalacturonan backbone and xylogalacturonan, rhamnogalacturonan I, and rhamnogalacturonan II regions. Majorly, pectin is present in processed foods as a thickening agent. It can also be used by pharmaceutical companies in the production of drugs. Pectin is one of the best fibres humans consume as it is edible and beneficial to the body. It is a binder that holds cells, tissues and components together. Pectin has a major hold in the structure of cellulose and hemicellulose holding them firmly together to support the growth of the plant. Therefore, when consumed, it does not digest but binds the waste materials together. This is one of the reasons why food high in pectin when consumed enlarges the faecal waste, but give them a shape by binding them together. Lack of pectin causes irregularity in the faecal waste as nothing is holding them together. It is majorly found in fruits and vegetables and it can dissolve in water to form a gel that acts as a glue holding tissues or parts together. In the guts, this gel holds the waste materials together and softens their path in the excretory system i.e., colon. Due to its nature, pectin contains a minute quantity of calories, which enables its use in the manufacture of some confectionery and consumable products. It is used in preservatives and in other products such as yoghurt which as a by-product of milk needs a component that stabilizes the different components put together (Khubber et al., 2021). As a result of its binding properties, it can be used as a coating for medical products. It can also be used as a supplement as it can be added to food especially those deficient in fibre. The beneficial effect of pectin does not end in the excretory system, like all soluble fibre, pectin is a major soluble fibre that reduces the quantity of sugar in the blood and the same vein reduces the risk of diabetes. Its binding nature in the form of the gel helps to bind fat substitutes in the form of cholesterol together with the waste which is then excreted out of the body. Simply put, it reduces the risk of the body’s exposure to fatal diseases that might put the health at risk. Also, as a binder, it helps to absorb waste and other materials, thereby holding them tightly, hence, this characteristic ensures the binding of all the waste material to be pushed out preventing unforeseen growth that might lead to cancerous cells forming in the colon. It also prevents any stoppage in the flow of blood to the heart and vice versa. Not only does pectin reduce colon cancer, but it also destroys cancerous cells at the beginning stage. Pectin is the best food component to consume to reduce obesity and excess weight in the body. Fibre removes calories and cholesterol from the body. According to researches carried out on different animals, consuming pectin reduces fats in the body, thereby increasing the functioning of some hormones that might have been subsided due to fat in the body (Shanmugasundaram et al., 2017). Pectin is also a food for the bacteria in our stomach which prevents the eating up of the stomach by these same bacteria which can cause an ulcer, a gastrointestinal malfunctioning.

### 3.2 Processing of lignocellulose for industrial usage

The processing, also known as pre-treatment of lignocellulose is an important step to disrupt and expose the lignocellulosic matrix for the extraction of lignin, cellulose and hemicellulose and also, for structural modification. This is done to remove lignin/hemicellulose as well as cellulose decrystallization. The processing can either be through physical, chemical, physicochemical as well as biological pre-treatment (Alvira et al., 2010). Processing using physical pre-treatment involves mechanical comminution such as chopping, grinding and milling to reduce the particle size as well as cellulose crystallinity of the plant material. This is usually the initial step in the processing of lignocellulose. Other physical pre-treatment technologies are pulse-electric field (PEF) and pyrolysis. PEF involves the use of a high-intensity electric field which increase the permeability as well as the mechanical rupture of the plant cell wall, thereby, facilitating the access of acids and enzymes to breakdown cellulose. Pyrolysis, conversely, involves the decomposition of the plant material into a gaseous product at a very high temperature (>300 °C). Chemical pre-treatment involves the use of chemicals and the most commonly used chemical is dilute-acid hydrolysis. Dilute-acid hydrolysis solubilizes hemicellulose and can be done at high temperature (180 °C) for a shorter retention time, or at low temperature (120 °C) for longer retention usually between 30 to 90 min. Other chemical methods of lignocellulose pre-treatment include alkali hydrolysis which resulted in the removal of lignin and hemicellulose in parts thereby, increasing the accessibility of cellulose. Biological pretreat involves the usage of microbes such as white, brown and soft rot fungi capable of degrading wood. However, degrading wood using microorganisms presents some disadvantages which had led to the direct use of enzymes for the biological processing of lignocellulose (Peral, 2016).

## 4 Dietary fibre

Dietary fibre is said to be immune to the digestive enzymes, that is, the different enzymes present in the body cannot break them down as the body lacks the correct enzymes to break them down, so once consumed, they are excreted same way they are ingested. This would not have been possible if not for the beta-glycosidic bond linking the glucose molecules in both straight and branched chains. Dietary fibre includes cellulose and non-cellulose polysaccharides like pectin, mucilages, gums, hemicellulose, lignin and inulin (Theuwissen and Mensink, 2008). They serve as parts of plants that are processed as foods, but once ingested pass to the large intestines untouched, then down to the colon where they are excreted out of the body. Despite not being utilized by the digestive enzymes does not imply dietary fibre is completely useless. They perform different and important functions both in the digestive tracts and in the body as a whole. Dietary fibre can cause changes in how food is absorbed and broken down in the body, that is, they control the major activity of digestion in the body. From the time of the discovery of the health advantages associated with the consumption of fibre, many kinds of research have emerged on how to supplement it into our daily diets. Many food industries have developed their products by substituting the fat content with indigestible polysaccharides, as many foods with fibre recently in the world is considered to be of higher quality. They are used in several functional foods like meat products, beverages, drinks and beverages (Dhingra et al., 2012). Lately, fibre is being supplemented in drug formulations, helping to solve the issue of people living with allergic reactions. Though these supplements do not have the same effect as the natural fibre gotten from the plant as it has undergone processing and therefore contain some other component that is directly not fibre. Despite how primitive the consumption of fibre may seem; high consumption of fibre is also detrimental. Without enough water consumption, fibre may not digest, which means it will just form a bulk in the colon, which may eventually lead to constipation (Mayo, 2021).

### 4.1 Dietary fibre and its function in the body

There has been an association of dietary fibre with improvement in health reduction of diseases (Salovaara et al., 2007). Due to the nature of the human body, food is eaten and the waste associated with such foods is excreted out of the body after digestion. But recently, humans tend to consume foods that are processed and during processing, some chemicals are added to make better the quality of the food products and also enhance their shelf life. But a fact about these chemicals is that they store up in the body and may not likely be excreted since they are chemicals. They can also react with some organs in the body which can cause certain growth leading to diseases. Diseases do not just shoot up in the human body in a day, they are gradually formed competing with the immune system till they can overcome them and become visible to the body and termed as a disease. Once these diseases start exhibiting their symptoms, medications are taken to cure or subside their effects for a while, thus, activating the immune system again. But recent studies have proven that the foods human consume can bring about the growth of a disease that can result in death. This disease starts as growth, but in a hidden zone that will not be visible till it cannot be managed again. Naturally, it can be well said that dietary fibre improves our health and makes us live long as it helps in the prevention of some diseases which might have fatal side effects. It also improves and strengthens the functioning of digestive organs.

#### 4.1.1 Obesity

Recently, the rate of obesity has been on the high side which is because some of the foods humans consume are of high-fat content which digests to release more fat in the body which is however not safe. Dietary fibre rich foods have been linked to a reduction in body weight (Slavin, 2005). Humans tend to consume foods that are high in fats, present in the form of cholesterol, which is extremely dangerous to humans if it compiles up in the body for a long period, causing diseases such as obesity. Howarth et al. (2007) reported that the more fat and less fibre consumed, the higher the risk of obesity. Fibre absorbs water, including cholesterol that might be forming together to cause fetal growth in the body. It acts like a flusher to wash out the waste products that might want to bring about any growth which might lead to disease. Healthy consumption of dietary fibre reduces obesity.

#### 4.1.2 Satiety

Fibre has been indicated to increase satiation and lower calorie intake through consuming more volume of food, more chewing in addition to feeling of being full (Jimenez-Cruz et al., 2006), a reduction in micronutrients absorption, a slowing starch digestion rate, hormones and gut secretion alteration (Qi et al., 2006), an improvement in insulin sensitivity and an enhancement of the functionality of the pancreas (Liese et al., 2005).

#### 4.1.3 Improvement of diabetes

Another disease sprouting as a result of food humans consume is an increase in blood sugar levels. Dietary fibre has a positive impact on diabetic patients. It absorbs glucose, which prevents the excess discharge of glucose into the bloodstream hence, lowering the rate of glucose and converting this glucose to insulin, which is required in the restrain of blood sugar in diabetic patients. Studies carried out have shown an inverse relationship between dietary fibre and abnormal tolerance of glucose, the resistance of insulin in addition to the occurrence of syndromes of metabolism and type 2 diabetes (McKeown et al., 2004).

#### 4.1.4 Coronary heart disease

A high glycemic index is very dangerous and might cause or induce some diseases, such as stroke, hypertension and heart disease. Fibre has been shown to lower cholesterol.

#### 4.1.5 Gut health and gut cancer

Colon is known to be responsible for the passage and also storing of faecal before excretion. Storing of faecal increases the risk of waste storing up in the colon, which builds up into the growth of diseases. The likely disease that can shoot up from this growth is cancer which is one of the fatal diseases affecting mankind. Fibre is independently associated to a reduced risk of gut health and gut cancer (Bingham, 2006). Cancer was reported to be prevented due to intake of dietary fibre, water holding capacity and stool volume increased as a result of an increase in fibre intake and other toxins and carcinogens present in the column were diluted in the colon (Salovaara et al., 2007).

### 4.2 Classification of fibre based on their functions

Fibre is classified into three classes based on its function in the digestive system spread out to the rest of the body. These are;

#### 4.2.1 Bulking fibre

These types of fibre can come in two forms; either dissolvable in water (e.g., psyllium) or not dissolvable (e.g., cellulose and hemicellulose). They coagulate water to form a bulk (Colia, 2018), the bulk is then excreted out of the body due to the water coagulation which allows easy passage out of the body and also coagulates other waste materials out of the body. These fibres prolong a certain feeling of fullness of satisfaction after a meal which can reduce fat and also help in flushing out wastes from the body. Foods containing bulking fibre are vegetables such as broccoli, carrots, corn etc.

#### 4.2.2 Viscous fibre

This class of fibres functions as a thickener in the faecal mass (Kerns, 2018). When consumed, viscous fibre forms a gel-like substance that swallows up glucose and other food components, thereby reducing the rate at which they digest and also slowing down the blood sugar level increase. They include; beta-glucans, psyllium, pectins, glucomannan and guar gums. A good source of viscous fibre is asparagus, sweet potatoes etc.

#### 4.2.3 Fermentable fibre

These types of fibre induce the fermentation of foods into gases commonly known as “fart”. These occur in the large intestines as the bacteria present there utilizes the energy from short-chain fatty acid to gases. Also, these fibres serve as food for the microorganisms found in the large intestines, which further produces fatty acids which are short-chain having different roles, promoting the health of the gastrointestinal tract. They include; guar, inulin, beta-glucans, pectins, oligofructose and beta-glucans (Adam et al., 2014).

### 4.3 Classification of dietary fibre (indigestible polysaccharide) based on their solubility

Based on solubility, fibre is categorized as soluble as well as insoluble dietary fibres (Table 2). Good sources of dietary fibre are cereal and cereal bran in cell wall formation.

**Table 2:** Dietary fibre.

Nutrients	Water solubility	Food additive	Linkage	Source/comments
Arabinoxylan (hemicellulose)	Soluble	Xylose residue; arabinose	β-(1,4)-linked ​xylose ​residues	Psyllium
Xanthan gum	Soluble	Glucose, mannose and glucuronic acid	β-(1,4), *α*-(1,3) and β-(1,2)	Produced by *Xanthomonas* bacteria from sugar substrates
Agar	Soluble	d-galactose and 3,6-anhydro-l-galactopyranose	α-(1→4)	Derived from red algae
Resistant starch	Non-soluble	Glucose	α-(1–4), β-(1–6)	Could be starch which is protected by shell or seed (type RS1), granular starch (type RS2) or retrograded starch (type RS3) High amylose corn, barley, high amylose wheat, legumes, raw bananas, cooked and cooled pasta and potatoes.
Inulin	Soluble	Fructose	β-(2-1)-d-frutosyl fructose	In different plants, e.g. topinambour, chicory, etc.

#### 4.3.1 Soluble fibre

Soluble dietary fibre act like a magnet that attracts water into the digestive tract from the body (Kate, 2020). Water solubilizes this soluble fibre in the gut to form a gel which eases the passage of food (Cheng et al., 2017). Indigestible polysaccharides that fall in this category include xanthan gum, arabinoxylan, pectin, agar and inulin. These fibres once they arrive in the large intestine undergo the process of fermentation by the bacteria present there. The bacteria use short-chain fatty acid as energy to ferment these fibres into nutrients to benefit and strengthen the health of the digestive system. Psyllium is a soluble fibre but does not undergo fermentation rather it increases the quantity of excreta passed out of the body. This soluble fibre benefits the digestive tract as its fermentation serves as a source of food for the microorganisms in the stomach. The gel formed by soluble fibre also helps in the removal of cholesterol, as it is in the form of fat. This cholesterol is washed off as the fibre absorbs the water, thereby reducing the cholesterol level in the body as it will be utilized in the small intestine. Also, it is recommendable for diabetic patients as it has a longer full effect than most foods. Surampudi et al. (2016) reported the health benefits of consuming soluble dietary fibre which include lowering blood pressure, reducing inflammation and lipid level, improving immune functions, weight loss in addition to blood glucose control. The sources of soluble fibre include; vegetables such as Jerusalem artichokes, broccoli, carrots, tubers and root vegetables such as sweet potatoes, onions, husks of psyllium seed (a mucilage soluble fibre), flax seeds, nuts with almonds being the highest in dietary fibre, flax seed, oats, rye, chia and barley.

#### 4.3.2 Insoluble fibre

Insoluble fibre refers to the category of fibre that are not soluble in water and it includes cellulose, hemicellulose, resistant starch, lignin, etc (Table 2). They do not allow a situation whereby one is unable to pass out waste materials because they do not dissolve in water, they are therefore passed out of the body by increasing the bulkiness of faecal (Cummings, 2001). Insoluble fibre adds pressure to the bowel, helping to push out faecal mass. This cannot be achieved without adequate moisture intake; hence, psyllium now comes into action by supplying enough moisture for the passage of food. Some foods that contain fibre (insoluble) are wheat and corn bran, whole-grain foods, nuts and seeds, potato skins, lignans, fruits such as avocado and unripe bananas, peels of some fruits including kiwifruit, grapes and tomatoes.

### 4.4 Xanthan gum

Xanthan gum is an exocellular heteropolysaccharide produced by *Xanthomonas campestris*, a gram-negative bacteria and is an essential biopolymer used industrially (Garcia-Ochoa and Casas, 2000). It is a water-soluble dietary fibre and has a primary structure that consists of repeating units of pentasaccharides formed through two units of glucose, 2 units of mannose and one unit of glucuronic acid in the ratio of 2:2:1. Structurally, the glucose moieties of the polysaccharide are linked in a β-(1,4) glycosidic linkage while the mannose moieties are linked innerly to glucose moieties in an α-(1,3) configuration, generally to alternate glucose moieties. The d-glucuronic acid moieties are linked in a β-(1,2) configuration to the inner mannose moieties. The outer mannose moieties are linked to the glucuronic acid moieties in a β-(1,4) configuration. Some of the many industrial applications of xanthan gum include its use in salad dressings, dairy products, syrups, baked goods, dry mixes, frozen foods and beverages. Other industrial applications of the gum are its use in cosmetics, formulation of pharmaceuticals and slurry explosives, printing pastes for the textile industry, rust removers, and agricultural products. It is extremely soluble in hot and cold water (Sworn, 2021), a property attributed to the nature of the polyelectrolyte of its molecule. The solutions of xanthan are high in viscosity even when at low concentrations of polymer, a property conferring its use industrially particularly in food where it is effective as a thickening agent and in the stabilization of emulsions and suspensions (Petri, 2015). Its solutions have a pseudoplastic nature (Kumar et al., 2018) with its viscosity decreasing with an increase in shear rate and its viscosity also depending on temperature, on the concentration of biopolymer and salts and its pH. Xanthan gum has a moisture content of 8–15%, nitrogen content of 0.3–1%, an ash content of 7–12% and an acetate content of 1.9–6.0%. In its physical state, it is a dry powder that is cream in colour. Its viscosity is not affected by pH and its viscosity strongly increases with an increase in polymer concentration.

### 4.5 Agar

Agar is a heteropolysaccharide composed of agaropectin and agarose polymers. Typically, agar is composed of 70% agarose as well as 30% agaropectin. Agarose is a linear polysaccharide with no branched chain consisting a repeated galactose disaccharide. Agaropectin on the other hand varies in composition and is composed of D in addition to L isomers of galactose with sulfate and pyruvate substituents resulting in the polymer having a strong negative charge. It is a gelatinous material produced from red algae, especially *Gracilaria* species and it forms a part of the structural component of algae as well as other dietary fibre. These algae are generally Rhodophyta having red pigments and are sold in bricks, powder or flakes in translucent and amorphous forms. Naturally, agar exists as a cell wall component that is complex, which contains agarose (a polysaccharide) in addition to calcium and sulphate. It dissolves in boiling water and also has the general properties of soluble fibre, meaning that it acts as an absorbent. Due to its nature, it is often taken before a meal to cleanse the stomach and is also used in the laboratory as a component of solidification (medium for bacteriological culture) to test out microorganisms. Agar just like starch is a straight-chain and a branched-chain sugar. Also, just like all soluble fibres, it lengthens the duration of satisfaction as it takes time to digest and it also helps in the absorption of water to ease transportation of ingested food in the digestive system. It uses spread across different industries that utilize chemicals and rely on the growth of microorganisms. It is employed as a food additive such as a thickening agent in confectionery products (Kandale et al., 2011) such as desserts, ice-creams pastries, salad dressings and cake icing. They are also employed in canned poultry, meat and fish, in clarifying wine and beer. At 37 °C, agar solidifies to form a gel that comes out firm and at 42 °C, a dilute solution of agar remains liquid in boiling water.

### 4.6 Resistant starch

In healthy humans, resistant starch is starch as well as degrading products of starch, which are not digestible in their small intestine by enzymes (Olawoye et al., 2020a; Olawoye et al., 2020b). Several foods containing carbohydrates in different proportions have resistant starch. They are classified into four (4) subfractions which include physically inaccessible starch (RS_1_; also referred to as type I starch), native starch granules (RS_2_; also referred to as type II starch), retrograded starch (RS_3_; also referred to as type III starch) and chemically modified starch (RS_4_; also referred to as type IV starch) (Englyst et al., 1992). Their physical inaccessibility form is responsible for their resistant nature as found in partly milled seeds and grains in addition to some densely starchy foods that have been processed. Chemically, their measurement is determined as a disparity between released glucose produced by the digestive enzyme of a food sample that has been homogenized and boiled and the one released from a sample that is not boiled and homogenized (Olawoye and Gbadamosi, 2020a, Olawoye and Gbadamosi, 2020b). The heat stability of RS_1_ in the majority of normal cooking processes is responsible for its use extensively as ingredients in conventional foods (Sajilata et al., 2006). RS_2_ are starches exhibiting granular forms with resistivity to enzymic hydrolysis. Starch is tightly packed radially in raw starch granules and is dehydrated relatively. This closely packed structure restricts digestive enzyme accessibility, several amylases, which is the reason for the resistant quality of RS_2_ like starch that is not gelatinized (Sajilata et al., 2006). The most resistant fraction of starch is RS_3_ and is the main amylose that is retrograded when gelatinized starch is cooled. Hence, the majority of moist foods possess an indefinite quantity of RS_3_. Nevertheless, recurring cycles of heating as well as cooling elevates levels of RS_3_ in foods such as potatoes. Chemically, RS_3_ is measured as a small amount that resists dispersion via enzyme digestion and boiling as dispersion can only occur with dimethyl sulfoxide or KOH (Mudgil and Barak, 2013). RS_3_ completely resists digestion by the pancreatic amylases. RS_4_ is a chemically modified starch that has chemical bonds asides α-(1–4) or α-(1–6). A reduction in starch digestibility of the small intestine is a result of chemical modification hence RS_4_ formation. There may be a change in the starch content of foods during storage due to water content, temperature and during the preparation of food (Olawoye et al., 2022).

## 5 Inulin

Inulin consists of a linear fructose polymers mixture with various chain lengths together with a glucose molecule at each Carbon-2 end and connected via β-(2-1)-d-frutosyl fructose bonds. It is part of the fructan group of polysaccharides, which functions as a storage carbohydrate in many plant species. Inulin forms a part of a non-digestible polysaccharide known as fructans that is water-soluble. Majorly, it is utilized as a fat replacer, texture modifier, prebiotic, sugar replacer as well as for functional food development to enhance health as a result of its effective function in the health of gastric (Shoaib et al., 2016). Due to its availability in more than 300 vegetables, inulin is said to be distributed to a large extent in several types of plants and as a part of the human daily intake of food for many years, it contributes to significant nutritional and technological advantages. Inulin natural sources include Jerusalem artichoke, wheat, banana, asparagus, garlic, leek and onion. It is utilized in processed foods, giving desirable characteristics and in comparison, digestible polysaccharides give the energy of 25–35% only. Inulin is about 10% of the sweetness of sucrose in which they are prepared synthetically. It digests readily in the colon by bringing water into the colon for the management of constipation in addition to related ailments. It also helps in the promotion of microflora growth inside the digestive tract and is also considered a vital ingredient in the preparation of foods that are low in calories for the management of blood sugar levels in diabetic patients. Most inulin is produced commercially from chicory, but Jerusalem artichoke and dahlia are regarded as excellent sources for its production industrially in temperate regions. There are two (2) stages of inulin production. Firstly, raw syrup extraction and initial purification are carried out with further refining to produce commercialized products as the second stage. For obtaining a purified higher yield final product having a less consumption of energy during inulin extraction, some enhanced technologies such as ultrasound, supercritical carbon dioxide, pulse electric field and simultaneous ultrasonic/microwave are employed (Lou et al., 2009).

## 6 Food applications

The eye-opener to the advantages associated with consuming fibre has made it an enforced vital constituent in some food products. Prebiotics are substances that promote the growth of beneficial intestinal microorganisms which may be added to various food products. However, it must be noted that fibre can be prebiotic, though it is necessary to be aware of the fact that not all fibres are prebiotics (Samanta et al., 2015). Having carefully looked into the meaning of indigestible polysaccharides, their vital functions in our body, the process it undergoes in our body and the different classifications and types of indigestible polysaccharides present in our food, it is necessary to look into how the food industry has improvised these products into the various food products they manufacture for consumption. However, before the supplementation of a food product with dietary fibre, it must function like every other ingredient utilized in the production of the food and it must be supplementing the loss of a particular nutrient. For instance, it is usually supplemented to replace fats which can increase the level of cholesterol in the body. Due to their effective properties, they act on food and modify certain properties both physically and chemically. The different food industries that supplement indigestible polysaccharides include.

### 6.1 Bakery industries

In the baking industry, fibre has become a necessity as an ingredient and supplement to be used (Ktenioudaki and Gallagher, 2012). When a product is baked with fibre, it has an effect by extending the duration of its palatability. Soluble fibre absorbing water is also an advantage in baking as it can increase the weight of bread both after baking and after consumption. Also, fibre-rich bread is very good for marketing as it is good for the body and sick patients especially people living with diabetes. Lignocellulose and fibre-rich products, e.g., wheat bran, oat bran, potato peel from potato have been applied to replace wheat flour in the production of bread. In ready to eat breakfast foods and pastry products, fibre is used in their production process. A major reason for this supplement is that dietary fibre produces a wide variety of flavours for the products making it attractive and more palatable. Great importance is their fermenting abilities as they can ferment and give a better structure and a more attractive end product (Olawoye and Gbadamosi, 2020a, Olawoye and Gbadamosi, 2020b). The most important factor is that they are cost-efficient. Other products manufactured from flour e.g., noodles also have a supplement of dietary fibre added to it, but instead of the dietary fibre replacing the flour, it is only added as an additional ingredient. This is employed in places where noodles are one of their major sources of calories (Gbadamosi et al., 2020). The dietary fibre used for this product is soluble fibre because of its numerous benefits. The addition of dietary fibre brings about noodles with improved quality and quantity and also better palatability and it increases their shelf life (Bustos et al., 2015). In gluten-free bakery products, xanthan gum has been widely used as a gluten replacer for dough extensibility owing to the rigid structure of its molecules and a higher degree of solution pseudoplasticity.

### 6.2 Fibre enrichment

The dietary functions of some indigestible polysaccharides such as cellulose, hemicellulose, inulin, resistant starch as well as pectic substances avail them to be used as an essential ingredient in human diets. Such functions include nutritional substitutes as well as a good organoleptic characteristic. Being fibre constituents, indigestible carbohydrates enhance the texture and taste of food, especially in bakery products as well as breakfast cereals. Blanco Canalis et al. (2019) in his study reported an improvement in the properties of bakery and breakfast cereal when inulin was used compared to other fibres. The application of indigestible polysaccharides (fibres) to bakery and pastry products not only moistens and keep fresh the products for a long time but also resulted in crispiness improvement. Indigestible polysaccharides incorporated into cereal flour resulted in dietary fibre content increase, reduction in fat content and improved the incorporation of air during the mixing of the flour. Indigestible fibres that are soluble in an aqueous environment such as inulin, pectic substance, and xanthan gum has been incorporated into food products such as dairy products, drinks as well as a table spread.

### 6.3 As prebiotics in dairy products

Another way through which indigestible polysaccharides could be utilized is in dairy food products as prebiotics. Prebiotics are non-viable constituents of foods that are metabolized selectively by beneficial gastrointestinal bacterial which in turn modulates human guts and hence, confers a health benefit to the host (Olawoye et al., 2020). Human guts modulation by intestinal microflora can lead to the resistance of the guts against human pathogenic bacteria, reduce cancer risk, and lower blood ammonia and lipid concentration. One good indigestible polysaccharide that possesses prebiotic properties is inulin-type fructans. The degree of polymerization (DP) which represent the number of sugars (monosaccharides) present in the molecules is prominent in influencing the properties of dairy products such as sweetness, prebiotics activities, digestibility, textural as well as water-holding properties among others. Pimentel et al. (2013) during their research, utilized inulin as a fat replacer in low-fat yoghurt. It was observed that the inulin (long-chain oligofructose) formed microcrystals with the milk, which was not perceived orally when tasted but formed a fine creamy texture which promotes the oral sensation in comparison with yoghurt made of full-fat. The suitability of the long-chain inulin as a replacer of fat in dairy products was due to its stability, less solubility and high viscosity when compared with native inulin. In the work of Guimarães et al. (2018), different degree of inulin polymerization was used in the production of prebiotic whey beverage. It was reported that the physical stability of the whey beverage is significantly dependant on the degree of polymerization of inulin as higher DP of inulin resulted in higher physical stability of the beverage. Aside from the prebiotics effects of indigestible polysaccharides, it can be utilized to modify the textural attributes of dairy food products. De Castro et al. (2009) observed a change in the textural properties (pseudoplastic behaviour and consistency index value) of whole fat fermented milk with the incorporation of indigestible fibre. It was suggested that the textural modification of the fermented milk could be related to the effect of plasticizing of the fibre resulting in lower moisturizing as well as a decrease in the viscosity of the milk due to the reduction of the hydrodynamic volume of the milk protein. Also, Adding fibre to yoghurt is a brilliant concept, as when added it will absorb the excess water and therefore prolong yoghurt shelf life (Mohamed et al., 2014). Fibre also changes some characteristics in the yoghurt, for instance, fibre hardens the floppiness of yoghurt giving it a more solidified texture.

### 6.4 Meat industries

Meat, a highly versatile and nutritious food is the main source of protein in human diets. Aside from its high-water composition, meat is well-known for its high fat and protein content as it is derived from animals. To make meat and meat products healthier, health beneficial substances or ingredients can be added likewise, a substance that is considered to be detrimental or harmful to health can be eliminated or reduced. One valuable substance or ingredient that comes to mind from a health point of view is indigestible polysaccharides. Indigestible polysaccharides has been incorporated either solely or together with other ingredients in the formulation of low-fat meat products (either ground meat or meat emulsion). When used in meat products, indigestible polysaccharide helps to retain water, reduce cooking loss and maintain juiciness. A research carried out by Zhuang et al. (2016) on the addition of dietary fibre from sugarcane as well as emulsified sesame oil in the formulation of low-fat meat batter revealed that there was an improvement in the sensory scores and texture of the product. In other studies, Fernández-López et al. (2004) carried out the inclusion of lemon albedo and orange fibre flour; a soluble dietary fibre from citrus by-products on physicochemical and sensory properties of cooked and dry-cured sausages. The result obtained from the study revealed that the residual nitrite, which tended to induce the formation of nitrosamines was significantly reduced. One factor hindering the quality as well as shelf life of meat and its products is lipid oxidation. This is as a result of a higher number of unsaturated fatty acids and metal catalysts, ham pigments as well as oxidizing agents present in the meat tissues. Subjecting meat into various processing operations such as grinding, chopping, emulsification and flaking accelerated the onset of lipid oxidation through the liberation of the membrane-bound phospholipids. Hence, one good way to minimize or guide against the initial phase of lipid oxidation in meat and its products is through the inclusion or application of antioxidant indigestible polysaccharides. Owing to this, an indigestible polysaccharide with antioxidant properties from citrus and grape pomace, pineapple shell, mango peel and guava pulp had been reportedly used in mitigating against lipid peroxidation. Verma et al. (2013) as well as Das et al. (2015) in their studies, incorporated pectin from guava powder and pulp residue of bael in sheep meat nuggets and it was observed that lipid oxidation was inhibited in the meat nuggets at prolong storage compared to the control samples which received a lower sensory score (low flavour as well as odour) as a result of higher lipid oxidation. Also, dietary fibre from plant by-products had been reported to improve water and oil retention, stabilize both emulsion and oxidation as well as impart antimicrobial and anti-inflammatory activities of meat and meat products (Goñi and Hervert-Hernández, 2011).

### 6.5 Supplements

As technology advances, so does the researches on how to improve the fibre in the human diet. Recent researches have brought about taking fibre in the form of drug supplements or as an additive added to create a certain effect in food. These products are taken either to serve the purpose of the deficiency of fibre in the normal diet or to supply the lost nutrients during processing. The drugs and additives have the same effects as other multivitamins but they provide fibre instead. They are prescribed to people with health defects such as diabetics that require low sugar intake and also a high source of energy for their daily activities. It can also be prescribed for gastrointestinal disorders and other digestion problems (Papathanasopoulos and Camilleri, 2010). Resistant starch as a supplement helps in increasing the rate of insulin production in the blood, helps to promote ease of excretion and also builds up both the excretory and digestive system.

## 7 Conclusion

Indigestible polysaccharides are found in plant materials and when consumed in diet resist enzymatic digestion. These polysaccharides include lignocellulose, inulin, pectic substance, dietary fibre etc. They occur naturally in grains, fruit and vegetables and sometimes as by-products of agriculture and food processing. Their inclusion in diets confers health benefits such as prebiotics effects, lowering of blood ammonia and lipid concentration and reduced risk of cancer. Various indigestible polysaccharides from various sources had been explored by different researchers in food product formulation. Incorporating indigestible fibre into food products enhances their physical stability, consistency, rheological behaviour, textural and sensory properties as well as their suitability as functional foods. The addition of these polysaccharides into bread, breakfast cereals, cakes, cookies, beverages, meat and dairy products had been reported by researchers to yield a favourable result. Enriching food products with indigestible polysaccharides requires careful selection of the polysaccharide source as well as the method of incorporation. One future consideration or application of indigestible polysaccharides is it use as biodegradable packaging material due to environmental concern about polystyrene.

## Declarations

### Author contribution statement

All authors listed have significantly contributed to the development and the writing of this article.

### Funding statement

This research did not receive any specific grant from funding agencies in the public, commercial, or not-for-profit sectors.

### Data availability statement

Data will be made available on request.

### Declaration of interests statement

The authors declare no conflict of interest.

### Additional information

No additional information is available for this paper.
